# The role of polymorphic cytochrome P450 gene (CYP2B6) in B-chronic lymphocytic leukemia (B-CLL) incidence and outcome among Egyptian patients

**DOI:** 10.32604/or.2024.047021

**Published:** 2024-03-20

**Authors:** MENNA AL-ADL, MAGDY M. YOUSSEF, AHMED EL-SEBAIE, SHERIF REFAAT, AFAF EL-SAID

**Affiliations:** 1 Division of Biochemistry, Department of Chemistry, Faculty of Science, Mansoura University, Mansoura, 35511, Egypt; 2 Hematology Unit, Department of Clinical Pathology, Faculty of Medicine, Mansoura University, Mansoura, 35511, Egypt; 3 Medical Oncology Unit, Oncology Center Mansoura University, Mansoura, 35511, Egypt; 4 Department of Genetics, Mansoura University Children’s Hospital, Mansoura, 35511, Egypt

**Keywords:** B-CLL, Xenobiotics, Cytochromes P450, CYP2B6

## Abstract

Cytochromes P450 (CYPs) play a prominent role in catalyzing phase I xenobiotic biotransformation and account for about 75% of the total metabolism of commercially available drugs, including chemotherapeutics. The gene expression and enzyme activity of CYPs are variable between individuals, which subsequently leads to different patterns of susceptibility to carcinogenesis by genotoxic xenobiotics, as well as differences in the efficacy and toxicity of clinically used drugs. This research aimed to examine the presence of the CYP2B6*9 polymorphism and its possible association with the incidence of B-CLL in Egyptian patients, as well as the clinical outcome after receiving cyclophosphamide chemotherapy. DNA was isolated from whole blood samples of 100 *de novo* B-CLL cases and also from 100 sex- and age-matched healthy individuals. The presence of the CYP2B6*9 (G516T) polymorphism was examined by PCR-based allele specific amplification (ASA). Patients were further indicated for receiving chemotherapy, and then they were followed up. The CYP2B6*9 variant indicated a statistically significant higher risk of B-CLL under different genetic models, comprising allelic (T-allele *vs*. G-allele, OR = 4.8, *p* < 0.001) and dominant (GT + TT *vs*. GG, OR = 5.4, *p* < 0.001) models. Following cyclophosphamide chemotherapy, we found that the patients with variant genotypes (GT + TT) were less likely to achieve remission compared to those with the wild-type genotype (GG), with a response percentage of (37.5% *vs*. 83%, respectively). In conclusion, our findings showed that the CYP2B6*9 (G516T) polymorphism is associated with B-CLL susceptibility among Egyptian patients. This variant greatly affected the clinical outcome and can serve as a good therapeutic marker in predicting response to cyclophosphamide treatment.

## Introduction

B-chronic lymphocytic leukemia (B-CLL), the most widespread lymphoproliferative disorder affecting the elderly, is characterized by an aggressive expansion of monoclonal lymphocytes in the peripheral blood, bone marrow and lymphoid organs [1]. The global incident number of B-CLL was 1,034.67 ×10^2^ in 2019. The incidence varies by race and geographic location, which is higher among Caucasians while extremely low in Asian populations [2]. Even though the exact cause of B-CLL is unidentified, a combination of hereditary and environmental factors may contribute to the pathogenic pathways that lead to the development of the disease. For patients with B-CLL, chemotherapy remains the primary treatment option. The disease has a clinical trajectory marked by repeated recurrences and medication resistance, even if the majority of cases respond to first-line therapy [3].

Individuals are constantly exposed to high concentrations of environmental pollutants, drugs and toxins known as xenobiotics. If exposure to these xenobiotics continues, it may lead to genotoxicity due to disruptions in DNA integrity. On the other hand, one’s ability to biotransform harmful xenobiotics into harmless ones can be seen as a crucial defensive mechanism [4]. Cytochromes P450 (CYPs) are a superfamily of heam-containing enzymes catalyzing phase I biotransformation that metabolize different xenobiotics and endogenous compounds [5]. The expression of CYPs genes is affected by a unique combination of genetic and non-genetic factors that are responsible for the inter-individual variability in the enzyme’s catalytic activities; these factors include genetic polymorphisms, age, sex, hormones and disease state. Fifty-seven human cytochrome P450 enzymes have been identified and are classified into 18 families [6,7].

Cytochrome P450 2B6 (*CYP2B6*) is a crucial enzyme for the hepatic metabolism of fatty acids, steroids, and chemical detoxification. Consequently, endo- and xenobiotic metabolism may be affected by *CYP2B6* upregulation or inhibition [8]. Numerous commercially available medications, including ß-blockers, selective serotonin reuptake inhibitors, antivirals, antidepressants, analgesics, and anticancer drugs like cyclophosphamide, are also metabolized by *CYP2B6* [9].

An oxazaphosphorine prodrug called cyclophosphamide is widely used to treat CLL. A primary catalyst in the cyclophosphamide bioactivation pathways is *CYP2B6*. The varied pharmacokinetics of cyclophosphamide and its toxic byproducts, which can result in an inadequate response to therapy and the development of side effects that are clinically relevant, may be attributed to CYP-mediated metabolism [10].

The *CYP2B6* gene is a highly polymorphic gene that exhibits ~300-fold variability in expression, with many single nucleotide polymorphisms (SNPs) that have distinct ethnic frequencies and are linked to changes in enzyme activity and gene expression [11]. The liver is the primary site of *CYP2B6* expression; extrahepatic tissues such as the lungs, kidney, digestive system, and brain express it to a lower degree. *CYP2B6* constitutes ~6%–10% of the whole hepatic CYPs content and contributes to the metabolism of 10%–12% of commercially available drugs [9].

A commonly studied SNP, CYP2B6*9 (G516T, rs3745274), is characterized by the presence of a G to T transversion in exon 4 and is associated with decreased gene expression and reduced enzyme activity compared to the CYP2B6*1 wild-type allele [12]. The CYP2B6*9 variant was reported to be widely occurring, from ∼25% of whites to 60% of the Asian population, and has been revealed to affect pre-mRNA splicing, resulting in a diminished level of the normal mRNA transcript and a 50%–75% reduction in *CYP2B6* protein level [13]. This SNP may occur in combination with K262R in CYP2B6*6 or with other alleles (CYP2B6*7, *13, *19, *20, *26, *34, *36, *37 and *38). It can occur by itself in CYP2B6*9 as well [14].

CYP2B6*9 was previously reported to be associated with cancer risk. Justenhoven et al. concluded that the genetic variants CYP2B6*9, which are known to decrease activity of the *CYP2B6* enzyme, contribute to a higher risk of breast cancer [15]. This SNP was also found to be associated with hematological malignancies as well. Yuan et al. stated that the T-allele of the CYP2B6*9 SNP was associated with the risk of ALL and AML. They suggested that the inherited impaired function of the *CYP2B6* detoxification pathway may be an essential genetic determinant of leukemia risk [16]. Based on the fact that CYP2B6*9 enzyme has an important role in xenobiotic biotransformation and cyclophosphamide metabolism, we aimed to study the inter-individual variation of *CYP2B6* isoform among Egyptian B-CLL patients by detecting the presence of the CYP2B6*9 SNP and its possible association with the incidence and clinical outcome of the disease following cyclophosphamide chemotherapy.

## Materials and Methods

### Patients

Through the clinical presentation, bone marrow aspiration and immunophenotyping, the B-CLL diagnosis was performed. The current work is a case-control study with follow-up conducted on one hundred patients diagnosed with B-cell chronic lymphocytic leukemia at the Oncology Center Mansoura University (OCMU) in accordance with the National Comprehensive Cancer Network (NCCN) guidelines for B-cell chronic lymphocytic leukemia [17]. A control group of one hundred healthy volunteers was used. This research was carried out between March 2021 and February 2023 with consent from the Mansoura University Faculty of Medicine’s Ethics Committee (Code No. MD.21.03.62). All participants provided written informed consent, which was collected. Only patients with typical B-CLL were included. The exclusion criteria involved any person in the B-CLL and control groups who had a family history of cancer, atypical B-CLL patients or other causes of lymphoproliferative disease, typical B-CLL patients with co-morbidities such as renal insufficiency, hepatitis and heart diseases, or those with autoimmune anemia or thrombocytopenia, and B-CLL patients with a Tp53 deletion or mutation.

**Figure S1:**
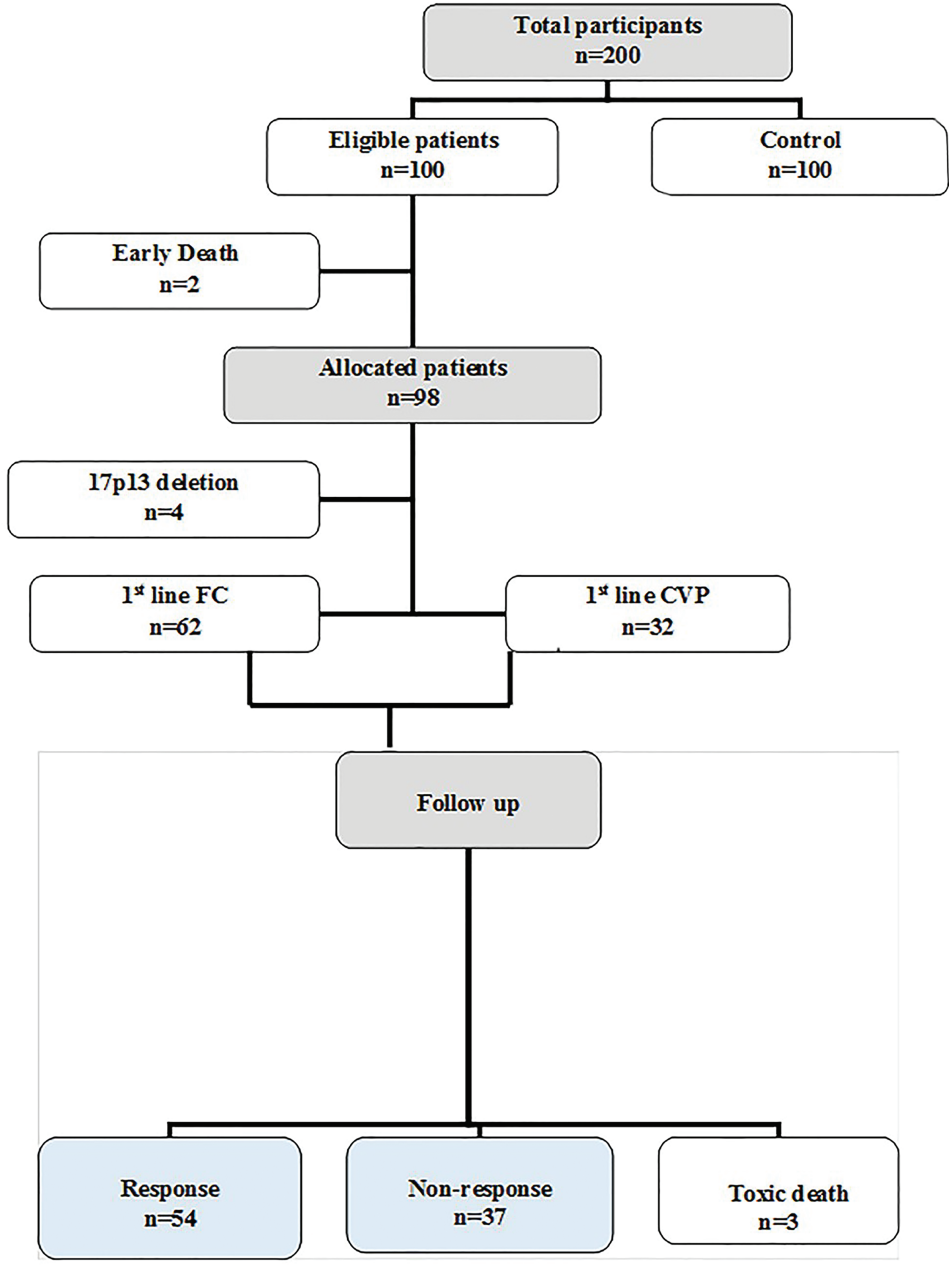
Flow diagram of the studied groups.

From the 100 eligible B-CLL patients, two patients died early and four patients with the 17p13 deletion were neglected. The other 94 allocated patients were classified into two groups according to the type of treatment received: [62 were treated with the Fludarabine/Cyclophosphamide (FC) regimen and 32 were treated with the Cyclophosphamide/Vincristine/Prednisone (CVP) regimen]. Patients treated with the FC regimen received it as follows: fludarabine (30 mg/m^2^) and cyclophosphamide (250 mg/m^2^), which were given i.v. on days 1–3 every 28 days, for a maximum of six courses. Patients treated with the CVP regimen received it as follows: cyclophosphamide (750 mg/m^2^) and vincristine (1.4 mg/m^2^ on day 1) given i.v. and oral prednisone (100 mg on days 1–5). This was repeated every 3 weeks, and this was continued for eight cycles. After three months since the last chemotherapy cycle, the bone marrow examination, radiological scan and blood tests were repeated. The treatment outcome of all the patients was determined according to the response criteria defined by the International Workshop on Chronic Lymphocytic Leukemia (IW-CLL), depending on the tumor load and recovery of bone marrow functions after chemotherapy [18] (Suppl. Fig. S1).

### Methods

Each participant in the B-CLL and control groups had six milliliters of blood drawn from them. Each blood sample was separated into two aliquots: 3 mL were placed in sterilized tubes containing EDTA for the hematological, genetic and flow cytometric investigations, whereas the other portion of the blood was left to clot, centrifuged at 4000 rounds per minute (rpm) for 10 min, and then serum was separated and stored at −20°C for the further biochemical measurements. Additionally, all B-CLL patients had their bone marrow aspirated, and the marrow smears were ready for the morphological analysis. For the cytogenetic study, two milliliters of the aspirate were put in heparin tubes.

### DNA extraction

From all participants, the genomic DNA was isolated from whole blood samples using a commercial extraction kit (QIAamp® DNA Extraction Kit, Cat. No. #51106, QIAGEN, Hilden, Germany), according to the manufacturer’s directives. After extraction, the DNA purity and concentration were determined by analyzing the A_260_/A _280_ and A_260_/A_230_ ratios using (NanoDrop™ 1000 Spectrophotometer, NanoDrop Tech., Inc., Wilmington, USA). Ten μL of DNA were added to 990 μL nuclease-free water in a quartz cuvette, and a reading was taken. The absorbances of proteins and nucleic acids are 260 and 280 nm, respectively. The purity of nucleic acid extraction has been measured using the ratio of absorbance at these wavelengths. “Pure” DNA is commonly defined as having a ratio of about 1.8. A high 260/280 ratio typically suggests that there is protein contamination in the sample. The predicted value of the 260/230 ratio, which is a secondary indicator of nucleic acid purity, is typically between 2.0 and 2.2. If the ratio is noticeably lower than anticipated, this indicates the presence of contaminants that absorb at 230 nm. After that, the DNA concentration was then computed by the formula: $c=\;\frac{A\times \epsilon }{b}$ , where C is the nucleic acid concentration in ng/μL, A is the absorbance, b (cm) is the path length, and ε is the extinction coefficient for dsDNA (ng × cm/μL). DNA concentrations below 80 ng/μl were excluded from further analysis.

### Amplification of CYP2B6*9 (G516T) variant

The genotyping and amplification of the CYP2B6*9 (G516T) variant were analyzed using allele specific amplification-PCR (ASA), previously described by Müller et al. The primers used were: (F1-wild-type: 5′-GACCCCACCTTCCTCTTCTAG-3′), (F2-variant: 5′-GACCCCACCTTCCTCTTCTAT-3′) and (R-common: 5′-GGTCATCCTTTTCTCGTGTG-3′). β-actin was used as a control gene, and the presence of the β-actin band was an indicator of successful PCR amplification (β-actin F: 5′-ATGTCCCCCGTCTGGCCTGG-3′) and (β-actin R: 5′-CTGTAGCCGCGCTCGGTGAG-3′) [19,20]. The PCR was applied using (SimpliAmp™ Thermal Cycler, ThermoFisher Scientific, Waltham, USA). Each PCR reaction was performed in two tubes, with a total volume of 25 μL for both. The first one contained 4 µL DNA extract (~100 ng/µL), 12.5 µL master mix (COSMO RED, Cat. No. #ND-1289-5O, Willowfort, Birmingham, UK), and 1 µL of each [F_1_, R, β-actin (F and R)] primer (10 pmol/µL). The second one contained 4 µL DNA extract, 12.5 µL master mix and 1 µL of each [F_2_, R, β-actin (F and R)] primer. The final volume in both reactions was reached with molecular-grade water. Every set of samples was run along with a negative control (containing all reaction components except for the genomic DNA).

The thermal cycler was used according to the cycling program consisting of initial denaturation (94°C for 5 min), 35 cycles of [denaturation (94°C for 30 s), annealing (53°C for 30 s), extension (72°C for 1 min)] and a final extension (72°C for 7 min). Every set of samples was run along with a negative control. Finally, the PCR products of CYP2B6*9 amplification (223 bp) and β-actin (321 bp) amplification were analyzed directly by agarose gel electrophoresis using 2.5% agarose gel and 1X Tris-borate-EDTA (TBE) buffer electrophoresis at 200 V for 30 min, stained with ethidium bromide (500 mg/L), and visualized under an ultraviolet trans-illuminator. The wild-type genotype (GG) appeared as one band at 223 bp in the 1^st^ PCR reaction, the variant genotype (TT) appeared as one band at 223 bp in the 2^nd^ PCR reaction and the heterozygous genotype (GT) appeared as two bands in both reactions at 223 bp.

### Immunophenotyping and cytogenetics

Immunophenotyping (IPT) was carried out using a flow cytometer (BD FacsCanto II ^TM^ Clinical Flow Cytometer System, BD Bioscience, New Jersey, USA). The chromosomal anomalies were identified by fluorescence *in-situ* hybridization (FISH). During the FISH cytogenetics analysis, dividing cells must have their chromosomes stopped at the metaphase stage using a spindle inhibitor, such as colchicine. The cells must then be hypotonically treated and fixed with Carnoy’s fixative (1:3 ratio of acetic acid to methanol) for the optimal period. After that, the methanol/acetic acid cells were placed onto glass slides that had been cleaned and given at least seven days to mature. Further, RNAse (100 µg/mL) was added to 2X standard saline citrate and incubated for an hour at 37°C. The slides were dehydrated using a succession of ethanol concentrations of 70%, 90%, and 99%.

Fluorescent-labeled DNA probes that hybridize to particular chromosomal areas were used to detect chromosomal abnormalities. A hybridization mixture containing ten microliters was overlaid on each slide, covered with a coverslip, and sealed with a rubber solution. The hybridized probe was detected with a fluorescein isothiocyanate (FITC)-conjugated avidin (Vector), and the fluorescence intensity was reinforced with a sandwich amplification with a biotinylated anti-avidin monoclonal antibody. The cells were counterstained with propidium iodide (2 µg/mL). The slides were evaluated under a fluorescence microscope (Olympus BX60 F5, Olympus, Tokyo, Japan). At least 200 nuclei were analyzed, and cells from three healthy donors with a normal karyotype were used as controls. Direct sequencing was used after the amplification of coding exons 4–10 and flanking intronic regions to investigate the mutational status of TP53 [21].

### Statistical analysis

STATA software version 17 was applied to calculate the sample size and the findings of Botros et al. when they examined the influence of CYPs polymorphisms on Egyptian patients with AML, as this is the first study to test CYPs polymorphisms in CLL patients from Egypt [22]. Analysis for a matched case-control study revealed that the sample size would be 47 participants per group (ratio 1:1), using a power of (80%) and an alpha error of (5%), and the chance of exposure in the control group would be 28%. To boost the study’s power, we used 100 cases and 100 controls in the current investigation. The Kolmogorov-Smirnov test was used to check for normality. The mean ± standard deviation (SD) was used to describe normally distributed data, the median (minimum and maximum) was used to describe non-normally distributed data, and percentages and numbers were used to represent qualitative data. The non-parametric data was processed using the Mann-Whitney and Kruskal-Wallis methods, while the parametric data was treated by the student *t*-test. Chi-Square was used to compare qualitative data between groups. The significance level was adjusted to (*p* < 0.05). The chi-squared (χ2) goodness-of-fit test was used to determine the SNP in both groups using the Hardy-Weinberg equilibrium equation. 95% confidence intervals (CI) for the odds ratio (OR) were obtained using binary logistic regression.

## Results

### Demographic data of the control and B-CLL groups

**Figure S2:**
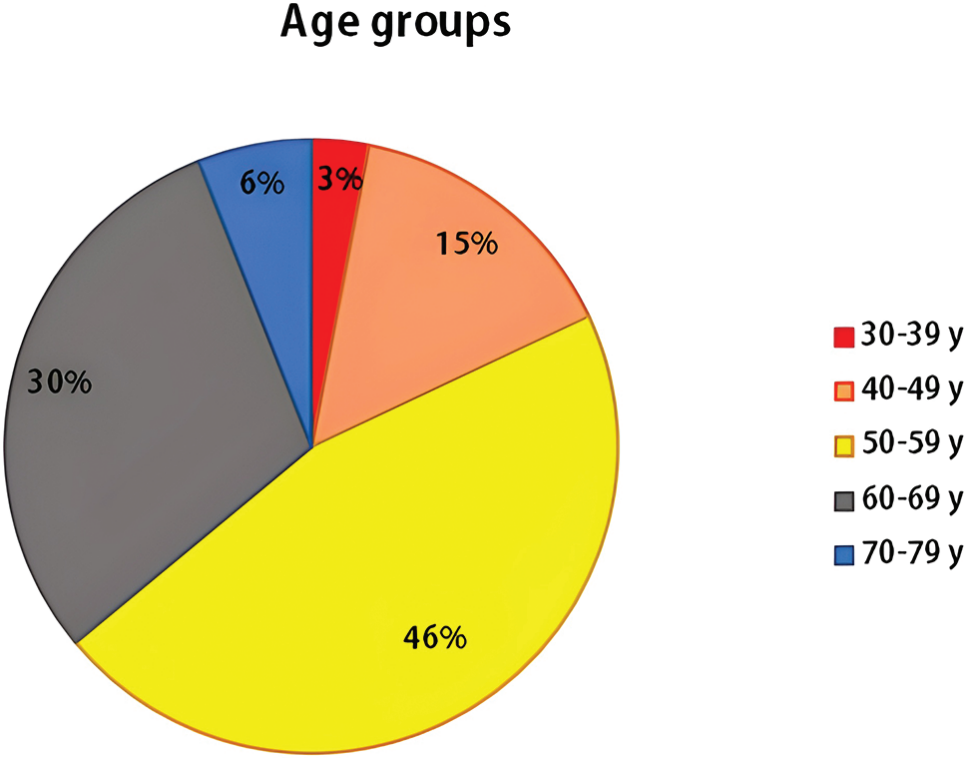
Spreading of B-CLL cases among various age groups.

The current work included 100 Egyptian B-CLL patients: 63% males and 37% females with a median age of 57.5 years, ranging from 36 to 77 years, and 100 age- and sex-matched healthy individuals (Suppl. Fig. S2). Regarding age and gender, we did not discover any appreciable differences between the two groups, as presented in Table 1.

**Table 1:** Demographic data of the control and B-CLL groups

Parameter	B-CLL		Control		*p*-value
**Age** (years)	*n* = 100		*n* = 100		
Median (range)	57.5 (36–77)		57.1 (35–74)		*p* = 0.74
Sex	*n*	%	*n*	%	
Male	63.0	63.0%	61.0	61.0%	*p* = 0.77
Female	37.0	37.0%	39.0	39.0%	

Note: Mann-Whitney U test and Chi-square test were applied.

### Clinical features and lab measurements of B-CLL patients

The IPT determined by the flow cytometric analysis of patients’ samples showed that (97%, 100%, 100%, 95%, 7%, 10%, 6%, 35%, 42%, 6%, 43% and 57%) of the patients expressed CD5, CD19, CD20, CD23, CD10, CD22, FMC7, CD38, CD49d, sIg, ƛ-light chain and ƛ-light chain, respectively. Cytogenetic analysis by FISH was determined successfully for all the patients and showed that 21%, 18%, 4% and 1% of patients presented with 13q14, 11q23 and 17p13 deletions and trisomy 12, respectively.

The physical examination and radiological scan showed that 100% of the cases presented with enlarged lymph nodes, 97% had splenomegaly and 86% had hepatomegaly. In addition, 12%, 16% and 17% of the patients presented with night sweats, weight loss and fever, respectively. The bone marrow aspiration revealed that 95% of patients presented with hypercellular marrow, and 5% of patients presented with normocellular marrow with mature lymphocyte infiltration. In addition, results of IGHV mutational status revealed that one patient carried an unmutated IGHV gene only.

Suppl. Table S1 demonstrated that the B-CLL group had significantly higher WBCs and absolute lymphocyte counts than the control (both were *p* < 0.001), but significantly lower absolute neutrophil counts, hemoglobin levels, and platelet counts (*p* < 0.001 for each). Furthermore, there was a significant rise in both the concentration of LDH and β2 microglobulin in the B-CLL group as compared to the control (both were *p* < 0.001).

Depending on whether a patient has lymphocytosis, lymphadenopathy, splenomegaly, hepatomegaly, thrombocytopenia, or anemia, the disease staging was determined using the Rai and Binet staging systems (Rai et al. 1975 [23]; Binet et al. 1981 [24]). We found that 59% and 41% of patients were classified as Binet B and C, respectively, based on the Binet staging, and 51%, 22%, and 27% of cases were classified as Rai II, III, and IV, based on the Rai staging. The International Prognostic Index-Chronic Lymphocytic Leukemia (IPI-CLL) was used to assess risk stratification according to a number of prognostic markers, including age, B-CLL stage, IGHV status, 17p13 deletion, and β2 microglobulin level (Eichhorst et al. 2021). We found that (10%, 79%, and 11%) of the cases were respectively stratified as low, intermediate, and high risk, respectively. The applied Rai and Binet staging systems depend on whether a patient has or does not have lymphocytosis, lymphadenopathy, splenomegaly, hepatomegaly, thrombocytopenia and anemia [23,24]. According to the Rai staging, 51%, 22% and 27% of cases were classified as Rai II, III, and IV, respectively, and according to the Binet staging, 59% and 41% of cases were classified as Binet B and C, respectively. The International Prognostic Index-Chronic Lymphocytic Leukemia (IPI-CLL) was used to assess risk stratification according to a number of prognostic markers, including age, B-CLL stage, IGHV status, 17p13 deletion, and β2 microglobulin level [25]. We discovered that the patients were categorized as low-, intermediate-, and high-risk groups in 10%, 79%, and 11% of the cases, respectively.

### Association of the CYP2B6*9 with the B-CLL risk

**Figure 1:**
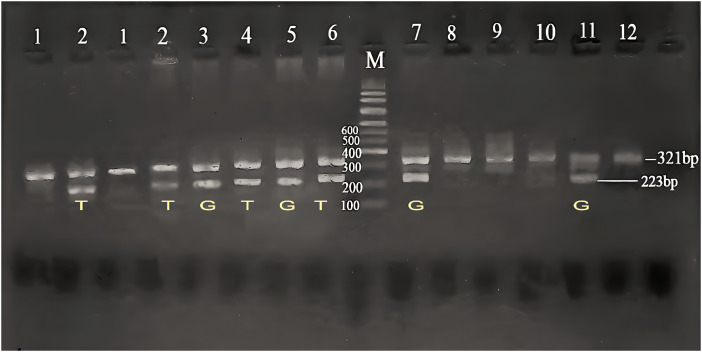
Agarose gel electrophoresis for *CYP2B6* (G516T) polymorphism in the studied groups. Lane (M) displayed the DNA marker; lanes 7,8,11,12 displayed (GG genotype at 223 bp); lanes 3,4,5,6 showed (GT genotype at 223 bp); lanes 1,2 displayed (TT genotype at 223 bp). The β-actin band was at 321 bp.

The *CYP2B6* gene polymorphism (*9, G516T) was investigated using ASA-PCR, and three genotypes: wild-type (GG), heterozygous carrier (GT), and homozygous variation (TT) were found. After being separated on an agarose gel, the PCR result was displayed in (Fig. 1).

**Figure S3:**
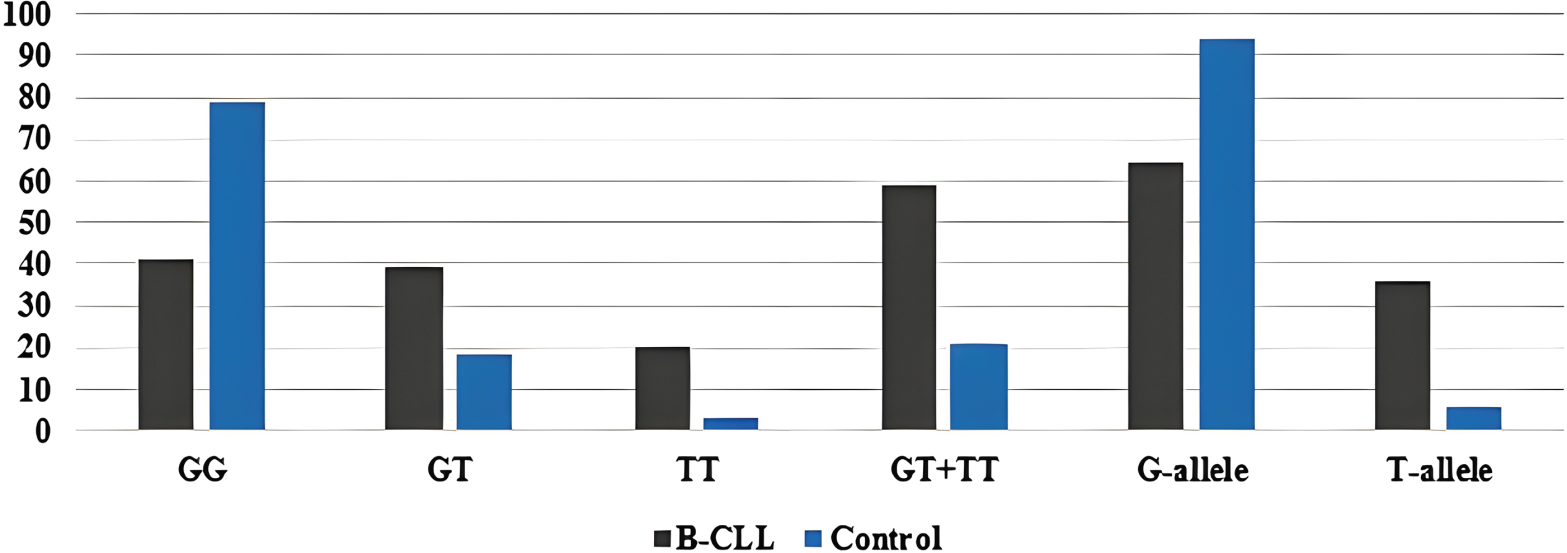
Genotypic and allelic frequencies of the CYP2B6*9 (G561T) SNP among the patients and controls.

The minor allele frequency of the T-allele was found to be 36% in patients and 6% in healthy controls. Furthermore, the common G-allele is shared by 64% of patients and 94% of healthy people. The frequency of the most common genotype (GG) was 41% in patients and 79% in healthy controls (Fig. S3). The genotypes of CYP2B6*9 in both the control and B-CLL groups were found to be in HWE by the chi-square test, meaning that there were no significant differences between the observed and predicted values (*p* = 0.14 and *p* = 0.06, respectively).

Combining various genetic association models (co-dominant, dominant, recessive, and allelic), B-CLL patients showed a strong link with the CYP2B6*9 (G516T) polymorphism. The co-dominant model (heterozygous comparison GT *vs*. GG, OR = 4.2, *p* < 0.001) and the homozygous comparison TT *vs*. GG, OR = 12.8, *p* < 0.001), the dominant model (GT + TT *vs*. GG, OR = 5.4, *p* < 0.001), the recessive model (TT *vs*. GG + GT, OR = 8.1, *p* < 0.001), and the allelic model (T-allele *vs*. G-allele, OR = 4.8, *p* < 0.001), as displayed in (Table 2).

**Table 2:** Genetic association models of CYP2B6*9 (G516T) variant and the risk of B-CLL

CYP2B6*9 (G516T)		B-CLL n (%)		Control n (%)		*p*-value	OR (95% CI)	
		n	%	n	%			
Genotypes	GG	41.0	41%	79.0	79%		Reference	
	GT	39.0	39%	18.0	18%	<0.001*	4.2	1.9– 9.3
	TT	20.0	20%	3.0	3%	<0.001*	12.8	3.5–45.6
Dominant model	GG	41.0	41%	79.0	79%		Reference	
	GT + TT	59.0	59%	21.0	21%	<0.001*	5.4	2.8–10
Recessive model	GG + GT	80.0	80%	97.0	97%		Reference	
	TT	20.0	20%	3.0	3%	<0.001*	8.1	2.2–28.3
Allelic model	G	128.0	64%	188.0	94%		Reference	
	T	72.0	36%	12.0	6%	<0.001*	4.8	2.8–7.9

Note: Chi-square test was applied. OR: Odds ratio, CI: Confidence Interval, *: Statistically significant (if *p* < 0.05).

### Association of the CYP2B6*9 with the characteristics of B-CLL patients

The wild-type (GG), heterozygous (GT), and variant (TT) genotypes for the *CYP2B6* (G516T) variant were evaluated to see if there were any differences in lab measurements and clinical characteristics, as indicated in (Table 3). The B-CLL patients in the (GT) and (TT) groups had a considerably lower median age than the (GG) group (*p* = 0.003 and *p* = 0.001, respectively). Gender-wise, there was a statistically significant difference in the proportion of male patients between the (GT) and (GG) groups (*p* = 0.009).

**Table 3:** Genotypic frequencies of CYP2B6*9 (G516T) variant stratified by clinical parameters and lab measurements among B-CLL patients

Parameter	GG (n = 41)	GT (n = 39)	TT (n = 20)	*p*-value	*p*-value within groups
Age (years)	60 (36–77)	54.5 (45–59)	52 (42–59)	*p* < 0.001*	*p*_1_ = 0.003*
					*p*_2_ = 0.001*
					*p*_3_ = 0.4
Sex (n%)					*p*_1_ = 0.009*
Male	20 (49%)	30 (77%)	13 (65%)	*p =* 0.09	*p*_2_ = 0.2
Female	21 (51%)	9 (23%)	7 (35%)		*p*_3_ = 0.32
CD38 (n%)					
Negative	27 (66%)	28 (72%)	10 (50%)	*p =* 0.78	_______
Positive	14 (34%)	11 (28%)	10 (50%)		
CD49d (n%)					
Negative	21 (51%)	22 (56.5%)	15 (75%)	*p =* 0.61	_______
Positive	20 (49%)	17 (43.5%)	5 (25%)		
Cytogenetics (n%)					
Normal	36 (88%)	19 (51.5%)	1 (10%)	*p <* 0.001*	
13q14 del.	3 (7%)	10 (23%)	8 (40%)		*p*_1_ = 0.01*
11q23 del.	1 (2.5%)	8 (20.5%)	9 (45%)		*p*_2_ < 0.001*
17p13 del.	0 (0%)	2 (5%)	2 (20%)		*p*_3_ = 0.04*
Trisomy 12	1 (2.5%)	0 (0%)	0 (0%)		
Binet stage (n%)					
B	28 (68%)	20 (51%)	11 (55%)	*p =* 0.81	_______
C	13 (32%)	19 (49%)	9 (45%)		
Rai stage (n%)					
ii	21 (51%)	16 (41%)	14 (70%)		
iii	9 (22%)	10 (25.5%)	3 (15%)	*p =* 1.0	_______
iv	11 (27%)	13 (33.5%)	3 (15%)		
Lymphocytes (×10^9^/L)	73 (51–95)	64.7(43–177.2)	68.1 (45.5–87.2)	*p* = 0.96	_______
β2 microglobulin (mg/L)	5.5 ± 1.86	5.6 ± 1.6	4.82 ± 2.1	*p* = 1.0	_______
LDH (U/L)	290 (188–401)	288 (180–395)	281.5 (191–381)	*p* = 1.0	_______

Note: Chi-square and Kruskal-Wallis tests were used. *: statistically significant (if *p* < 0.05), *p*_*1*_: comparison of GG *vs*. GT, *p*_*2*_: comparison of GG *vs*. TT, *p*_*3*_: comparison of GT *vs*. TT, LDH: Lactate dehydrogenase.

Additionally, the percentages of patients with chromosomal anomalies were greater in the (GT) and (TT) groups compared to the (GG) group (*p* = 0.01 and *p* < 0.001, respectively) and higher in the (TT) group than the (GT) group (*p* = 0.04). The CYP2B6*9 SNP did not significantly correlate with the illness stage, CD38 and CD49d expressions, absolute lymphocyte count, β2M, or LDH levels.

### Association of the CYP2B6*9 on the clinical outcome

We analyzed the genotypes of the wild-type (GG) and variant (GT + TT) to see if the *CYP2B6* (G516T) polymorphism had an impact on the clinical outcome after cyclophosphamide treatment. IW-CLL was used to evaluate the clinical outcome based on the tumor load and bone marrow function recovery. In contrast to patients with the GG genotype, who were classified as 83% responders, 14.5% non-responders, and 2.5%) no responders, patients carrying variant genotypes (GT + TT) were classified as (37.5% responders, 58.5% non-responders, and 4% toxic death). Based on the data in Table S2, we found that there was a significant (*p* < 0.001) association between the variant genotypes and response failure.

Results in Table 4A demonstrated that, with respect to the tumor load, the (GT + TT) group exhibited significantly higher mean percentages of lymphocytes infiltrating the bone marrow, absolute lymphocyte counts, and percentages of patients presenting with enlarged lymph nodes, liver, and spleen in comparison with the (GG) group (*p* < 0.001 for each). The cytotoxicity and bone marrow recovery results in Table 4B showed that the (GG) group’s absolute neutrophil count was significantly less than the (GT + TT) group’s. Furthermore, it was shown that patients in the (GG) group had more adverse events (*p* < 0.001) following chemotherapy than patients in the (GT + TT) group. These adverse events included fever episodes, hospitalization for longer than three days, infections, vomiting, and diarrhea.

**Table 4a:** Tumor load between patients with wild-type and variant genotypes concerning the CYP2B6*9 (G516T) variant following cyclophosphamide chemotherapy

Parameter		GG	GT + TT	*p*-value
		n = 40	n = 51	
Lymph nodes^♦^	Non-enlarged	32 (80%)	19 (37%)	<0.001*
	Enlarged	8 (20%)	32 (63%)	
Spleen^♦^	Non-enlarged	31 (77.5%)	20 (39%)	<0.001*
	Enlarged	9 (22.5%)	31 (61%)	
Liver	Non-enlarged	33 (82.5%)	20 (39%)	<0.001*
	Enlarged	7 (17.5%)	31 (61%)	
B-symptoms	Absent	37 (92.5%)	41 (80%)	=0.10
	Present	3 (7.5%)	10 (20%)	
Lymphocytes (×10^9^/L)	Median (range)	3.9 (0.5–165)	57.3 (30–177)	<0.001*
Marrow infiltration (%)	Median (range)	13 (4–88)	66 (42–89)	<0.001*

Note: Chi-square and Mann-Whitney U tests were used. *: statistically significant (if *p* < 0.05), ♦: enlarged lymph node if >1.5 cm and the enlarged spleen if >13 cm.

**Table 4b:** The cytotoxicity and bone marrow recovery between patients with wild-type and variant genotypes concerning the CYP2B6*9 (G516T) variant following cyclophosphamide chemotherapy

Parameter		GG	GT + TT	*p*-value
		n = 40	n = 51	
Hemoglobin (g/dl)	mean ± SD	11.1 ± 0.93	10.9 ± 1.03	=0.34
Neutrophils (×10^9^/L)	mean ± SD	2.48 ± 1.29	3.49 ± 1.14	<0.001*
Platelets (×10^9^/L)	median (range)	131 (60–220)	155 (50–211)	=0.14
Adverse events^♦^	Absent	16 (40%)	45 (88%)	<0.001*
	Present	24 (60%)	6 (12%)	

Note: Student-*t* test, Mann-Whitney U test and Chi-square test were applied. *: statistically significant (if *p* < 0.05), Adverse events ^♦^: febrile fever, admission to hospital >3 days, vomiting, diarrhea, or infections.

## Discussion

B-chronic lymphocytic leukemia (B-CLL) is a chronic lymphoproliferative disorder characterized by monoclonal B-cell proliferation [26,27]. B-CLL can manifest as an aggressive, fatal form of the disease or as an indolent variant that does not need to be treated for many years. Although the currently existing clinical staging techniques for B-CLL are simple and inexpensive, they are not accurate enough to forecast the course of the disease and a patient’s prognosis [28].

Chemotherapy responses across and within B-CLL patients vary widely, and this variability is unknown for any given patient. It has been proposed that variations in chemotherapy metabolism may be involved in controlling responses over time, both between and within patients [25]. Genetic polymorphisms in important drug-metabolizing enzymes such as cytochromes p450 (CYPs) have been emphasized as one of the main causes of variability in drug responses. As the liver is the main site of drugs’ metabolism, changes in the activities or expressions of drug metabolizing enzymes will have the potential to alter hepatic clearance and drug bioavailability [29].

This study aimed to investigate the potential correlation between the CYP2B6*9 (G516T, rs3745274) SNP and B-CLL susceptibility, as well as the effect of this connection on the clinical outcome after cyclophosphamide treatment among Egyptians. Our population is a perfect genetic model for conducting this kind of research because of its relative genetic homogeneity without inter-ethnic variations. The process through which populations of organisms alter over many generations is known as evolution. Genetic variations underlie these changes. Genetic variations can result from normal processes such as genetic recombination, which is the rearranging of genetic material as a cell prepares to divide, or from gene variants, often known as mutations. Different traits can be introduced into an organism by genetic variations that change the function of proteins or gene activity. Natural selection refers to the process wherein a genetic variant is more likely to be passed down to the following generation if the characteristic is favorable and helps the individual survive and reproduce. As generations of individuals with the trait continue to reproduce, the advantageous trait becomes more prevalent in a population over time, making the population different from an ancestral one. We particularly chose to investigate the CYP2B6*9 variant allele because of its high frequency and its recognized effect on the decrease in *CYP2B6* expression and function.

The B-CLL diagnosis was performed based on the diagnostic criteria of NCCN guidelines for B-chronic lymphocytic leukemia and the World Health Organization (WHO) classification. A typical immunophenotyping of monoclonal B-cells with a B-CLL score (4–5), <55% prolymphocytes-like cells in the peripheral blood smear and >5 × 10^9^/L absolute mature lymphocytes were the main characteristics of the patients [30,17]. B-CLL cells highly express CD5, CD19, and CD23 in addition to a single light chain expression (κ or λ), supporting the monoclonality of B-cells. Additional markers may also have been identified, such as CD10 and CD200, which help in the differential identification of suspicious cases, and CD38 and CD49d, which have prognostic value in B-CLL [31]. Using flow cytometry, we discovered that the expression of (CD5, CD19, CD20, CD23, CD10, CD22, FMC7, CD38, CD49d, sIg, ƛ-light chain and ƙ-light chain) was present in (97%, 100%, 100%, 95%, 7%, 10%, 6%, 35%, 42%, 6%, 43% and 57%, respectively). Expression of CD38 and CD49d is thought to be a poor prognostic factor as they help leukemia cells migrate and homing to secondary lymphoid organs and stimulate B-CLL cell proliferation in tandem with BCR signaling [32].

Cytogenetic analysis is useful for identifying certain chromosomal abnormalities that affect the disease’s prognosis and could affect the choice of treatment [33]. We observed that 1%, 18%, 4% and 1% of patients, respectively, had 13q14 del, 11q23 del, 17p13 del and trisomy. Only one patient had unmutated (IGHV-UM). B-cells with IGHV-UM are generally thought to be poor prognostic markers since they are highly susceptible to antigenic stimulation and are correlated with a lower survival time [34]. Leukemic B-cells are thought to constitutively secrete β2 microglobulin (β2M), and the tumor burden is correlated with the serum levels of β2M. A serum level of β2M is used in determining the risk rank of B-CLL patients, and β2M >3.5 mg/L is also considered a bad prognostic marker [25].

Leukemic cells invade the lymphoid organs and bone marrow as the disease worsens. Before and after starting cytotoxic agent treatment, bone marrow examination gives oncologists the information they need to determine if cytopenias are caused by leukemic infiltration or treatment-related myelo-suppression. The lab results showed that the patients had considerably greater WBCs and absolute lymphocyte counts than the controls (*p* < 0.001 for both). Absolute neutrophil and platelet counts in the patients were substantially lower than in the controls (both with *p* < 0.001). Likewise, the patients’ Hb level was considerably lower than that of the control group (*p* < 0.001). This may be explained by the fact that megakaryocytes, myeloid precursors, and erythroid precursors are all reduced concurrently with lymphocytic infiltration of the bone marrow [18]. Serum LDH levels, which indicate the disease’s proliferative load, were higher in patients than in controls (*p* < 0.001) which could be explained by the related tumor lysis syndrome, which is brought on by the disintegration of leukemic cells [35].

Analysis of CYP2B6*9 polymorphism showed that the patients displayed greater percentages of (GT) and (TT) genotypes than controls (*p* < 0.001 for both), carrying a 4- and 13-fold risk of developing B-CLL [(OR = 4.2, 95% CI 1.9–9.3) and (OR = 12.8, 95% CI 3.5–45.6), respectively]. Additionally, patients had a variant T-allele frequency that was substantially greater than that of controls (*p* < 0.001), with an estimated 5-fold increased risk of B-CLL (OR = 4.8, 95% CI 2.8–7.9).

Several studies provide evidence for the pathogenic role of the *CYP2B6* (G516T) polymorphism in the susceptibility to hematological malignancies. Yuan et al. have reported that the T-allele could be one of the risk factors for ALL and AML predispositions. They found that GT and GT + TT genotype frequencies were greater in ALL (37.5% and 42.7%, respectively, *p* < 0.01), and AML (37.2% and 40.9%, respectively, *p* < 0.01) than in control (23.9% and 25.9%, respectively), suggesting that the inherited defective function of the *CYP2B6* detoxification pathway may be an important genetic determinant of leukemia risk [16]. Daraki et al. also informed us that the T-allele was related to AML; higher frequencies of the variant genotypes (GT and TT) were found in cases compared to controls (GT: 38.8% *vs*. 29.8% and TT: 9.3% *vs*. 5.3%, respectively, *p* = 0.001) [36]. Berköz et al. informed us that the risk of acute leukemia development was 2.48-fold for GT genotype carriers and 1.92-fold for T-allele carriers [37]. In Egypt, Botros et al. reported that the *CYP2B6* gene polymorphism carries a 3-fold risk of AML [22]. No study has examined the impact of the *CYP2B6* (G516T) polymorphism on the incidence of CLL; therefore, it is quite demanding to anticipate the effective role of these genotypes in CLL risk.

When age at disease diagnosis was considered in our study, it was found that the B-CLL patients were distributed in all age groups (30–39, 40–49, 50–59, 60–69 and 70–79 years), but the highest frequency of patients (46%) was found in the (50–59 years) group. It was previously known that the risk of B-CLL development rises with advanced age, with a median age at disease onset of 70 years old [38].

Upon examining the association of the CYP2B6*9 (G516T) polymorphism with B-CLL, we observed that the median age of the disease onset was significantly lower in the (GT) and (TT) groups than the (GG) group (*p* < 0.001). Individuals with variant genotypes (GT) and (TT) have less metabolic activity of the *CYP2B6* enzyme. Absence or lowering the *CYP2B6* enzyme activity leads to improper metabolism, resulting in a carcinogenic effect, which might be responsible for an earlier onset of disease.

Given that men are more likely than women to acquire CLL, we investigated if there might be a relationship between gender and the *CYP2B6* (G516T) polymorphism. We discovered that the percentage of the (GT) genotype’s carrier was higher in males than females (*p* = 0.009). The regulatory region of *CYP2B6* has an estrogen-responsive element, and it has recently been shown that females have 1.7 times higher enzyme activity than males. This implies that males may be more susceptible than females to the development of such lymphoproliferative disorders due to the *CYP2B6* enzyme’s declining activity [39].

Oxidative stress products induce *CYP2B6*, which is a defense mechanism against DNA damage that may aid in leukemogenesis. The T-allele is associated with decreased enzymatic activity as well as a decreased capacity to metabolize and render numerous carcinogenic substances inactive, including naphthalene, aflatoxin B1, benzene metabolites, trichloroethylene, and alkylating agents. This could imply that those who are homozygous or heterozygous (GT or TT) are unable to metabolize genotoxic substances effectively, which causes cellular damage to accumulate. When considering the aforementioned information along with the higher frequency of (GT) and (TT) genotypes observed in patients with chromosomal aberrations (*p* < 0.001), one could hypothesize that *CYP2B6* enzyme deficiency could influence a person’s susceptibility to heamatotoxic exposure to leukemogens and could raise the risk of developing B-CLL.

For individuals with B-CLL, chemotherapy remains the cornerstone of current frontline treatment. Alkylating agents, like chlorambucil and cyclophosphamide, and purine analogues, like fludarabine, are the two main groups of chemotherapeutic medications employed [40]. For B-CLL patients lacking a Tp53 mutation or 17p deletion, cyclophosphamide (CPA) therapy remains the optimal option [41]. According to the patient’s age and fitness, CPA is typically used in combination with other medications like FC (fludarabine + cyclophosphamide) and CVP (cyclophosphamide + vincristine + prednisone). Despite the fact that most patients benefit after the first treatment, B-CLL is still an incurable condition with a clinical course marked by recurrent episodes that eventually lead to chemotherapy resistance. Individual patients differ significantly in their growth dynamics, response to treatment, and tendency for disease acceleration and transformation. Some biological characteristics have been identified as being linked to unfavorable outcomes. These variables include advanced disease stage, male gender, older age, β2 microglobulin level, IGHV gene status, TP53 mutation or deletion, and 11q chromosomal deletion [25]. TP53 continues to be the most highly correlated factor with chemoresistance; yet, not all patients who did not experience a response may be explained by this factor. As such, we ought to take into account other factors that could influence the pharmacokinetics or pharmacodynamics of the chemotherapeutic drugs.

*CYP2B6* is an essential enzyme involved in the conversion of CPA to an active form responsible for its anticancer activity. CPA is a prodrug that requires metabolic activation to produce its cytotoxic metabolite. The oxidation of CPA to create 4-hydroxy-CPA (4-OH-CPA), which is catalyzed by numerous members of the cytochrome P450 enzyme superfamily, including *CYP2B6*, is a crucial step in the bioactivation of CPA. This unstable intermediate binds to erythrocytes before being delivered to the tumor tissue, where it undergoes a spontaneous process of elimination to produce phosphoramide mustard and acrolein. The cytotoxic effects of phosphoramide mustard are caused by the cross-linking of DNA strands that occurs when it spontaneously cyclizes into aziridinium ions [42]. The most striking finding in our work is the relationship between the CYP2B6*9 (G516T) polymorphism and the failure to respond to CPA treatment. We found that the presence of the T-allele was associated with a significantly lower response rate in treated patients, unlike patients who had the normal G-allele (*p* < 0.001). Patients with the wild-type genotype (GG) were (83% responders, 14.5% non-responders and 2.5% toxic deaths), in contrast to the patients with variant genotypes (GT + TT), who were (37.5% responders, 58.5% non-responders, and 4% toxic deaths). This observation could be explained by the fact that the presence of the T-allele is associated with lower *CYP2B6* gene expression and enzyme activity [12].

The restoration of hemopoietic function is one of the criteria used to define remission. Therefore, rather than reflecting worse cytoreduction, it is probable that the decreased response rate seen in patients with the variant genotypes actually reflects higher hematopoietic toxicity; care was taken to ensure that this impact was frank. This option was ruled out by the greater post-treatment neutrophil count and higher residual tumor burden (lymphocytosis, lymphadenopathy, and hepatosplenomegaly) seen in the T-allele patients (*p* < 0.001 for each). It was of interest to determine whether this effect extended to treatment toxicity as evidenced by documented adverse events that are indicative of the toxicity of alkylating agents, including days in the hospital, febrile episodes, nausea and vomiting and diarrhea.

To address this question, patients with wild-type and variant genotypes were compared for the frequency of these adverse events determined after receiving chemotherapy, and we found that patients with the variant genotypes had fewer adverse events (*p* < 0.001). All of these data point to a strong possibility that the T-allele reduces the effectiveness and toxicity of CPA by interfering with its conversion to its active form.

Our results were in concordance with the Shu et al. findings [43]. They showed that CYP2B6*9 encoded an enzyme with low metabolic activity in CPA 4-hydroxylation *in vivo* and *in vitro*. The in-vitro findings confirmed that the T-allele confers lower mRNA expression and slower CPA 4-hydroxylation, indicating that its carriers are poor CPA metabolizers. Furthermore, Johnson et al. found that individuals with at least one *6 allele had a lower chance of achieving a full response to FC; this suggests that the CYP2B6*6 allele is an independent predictor of a worse response to FC. A higher probability of not responding to the FC protocol was associated with the *6 allele (OR = 4.375, 95% CI: 1.046–18.265, *p* = 0.042). According to their findings, CYP2B6*6 is associated with reduced CPA efficacy, fewer treatment-related side effects, and a higher chance of FC chemotherapeutic response failure in CLL patients [44]. To our knowledge, though, this SNP has not been examined in CLL patients in any other reports outside of the Johnson et al.’s study.

The study’s primary constraint is the comparatively small sample size of the patient and control groups. Even though the findings might have therapeutic implications, more instances in the study are needed to validate our data. To validate our results and determine the prevalence of this SNP in other populations while taking ethnic heterogeneity into account, more research is advised. Furthermore, to fully comprehend the potential of the *CYP2B6* candidate gene for cancer susceptibility, additional research, including large sample sizes, gene-gene, and gene-environment interactions, is needed.

In conclusion, our research indicates that the CYP2B6*9 polymorphism plays a pathogenic role in the predisposition to B-CLL. This implies that inherited malfunction of the *CYP2B6* detoxification pathway could be a crucial genetic factor influencing the likelihood of developing B-CLL. Furthermore, the findings point to *CYP2B6* genotyping as a desirable candidate marker for routine clinical application and a step toward personalized medicine to get the most effective course of treatment.

## Supplementary Materials

**Table S1:** Lab measurements of patients and controls

Parameter	B-CLL (n = 100)	Control (n = 100)	*p*-value
WBCs (×10^9^/L)	75.2 (51–180)	7.8 (5.1–9.9)	<0.001*
Lymphocytes (×10^9^/L)	69.2 (43–177.2)	2.02 (0.86–4.01)	<0.001*
Neutrophils (×10^9^/L)	3.7 ± 1.41	5.6 ± 0.93	<0.001*
Platelets (×10^9^/L)	197.2 (80–269)	274.1 (220–322)	<0.001*
Hemoglobin (g/dl)	10.98 ± 1.21	13.01 ± 0.87	<0.001*
LDH (U/L)	289.5 (180–401)	180.8 (106–211)	<0.001*
β2 microglobulin (mg/L)	5.09 ± 1.99	1.64 ± 0.59	<0.001*

Note: Student *t*-test and Mann-Whitney U test were applied. *: Statistically significant (if *p* < 0.05), WBCs: White blood cells, LDH: Lactate dehydrogenase.

**Table S2:** Correlation between CYP2B6*9 (G516T) variant and the clinical outcome following cyclophosphamide chemotherapy

Outcome	Wild-type genotype (GG)	Variant genotypes (GT + TT)	*p*-value
	n = 41	n = 53	
Response	34 (83%)	20 (37.5%)	<0.001*
Non-response	6 (14.5%)	31 (58.5%)	<0.001*
Toxic–Death	1 (2.5%)	2 (4%)	= 0.71

Note: Chi-square and Fischer Exact tests were applied. *: Statistically significant test (if *p* < 0.05).

## Data Availability

The dataset utilized in the preparation of this study will be available from the corresponding author upon reasonable request.

## Abbreviations

λ-light chain: Lambda-light chain
κ-light chain: Kappa-light chain
ALL: Acute lymphoblastic leukemia
AML: Acute myeloblastic leukemia
ALT: Alanine transaminase
ASA: Allele specific amplification
AST: Aspartate transaminase
B-CLL: B-chronic lymphocytic leukemia
BCR: B-cell receptor
CBC: Complete blood count
CPA: Cyclophosphamide
CVP: Cyclophosphamide/Vincristine/Prednisone
CYP2B6: Cytochrome P450, family 2, subfamily B, member 6
CYPs: Cytochromes P450 superfamily
EDTA: Ethylene diamine tetra acetic acid
FC: Fludarabine/Cyclophosphamide
FISH: Fluorescence *in situ* hybridization
Hb: Hemoglobin
IGHV-M: Mutated immunoglobulin heavy chain variable
IGHV-UM: Unmutated immunoglobulin heavy chain variable
IPT: Immunophenotyping
IW-CLL: International Workshop on CLL
LDH: Lactate dehydrogenase
NCCN: National Comprehensive Cancer Network
NF-κB: Nuclear factor kappa B
OCMU: Oncology Center Mansoura University
PCR: Polymerase chain reaction
sIg: Surface immunoglobulin
SNP: Single nucleotide polymorphism
TBE: Tris-borate-EDTA
WBCs: White blood cells
β2M: Beta-2 microglobulin
